# Epigenetic mediated zinc finger protein 671 downregulation promotes cell proliferation and tumorigenicity in nasopharyngeal carcinoma by inhibiting cell cycle arrest

**DOI:** 10.1186/s13046-017-0621-2

**Published:** 2017-10-19

**Authors:** Jian Zhang, Xin Wen, Na Liu, Ying-Qin Li, Xin-Ran Tang, Ya-Qin Wang, Qing-Mei He, Xiao-Jing Yang, Pan-Pan Zhang, Jun Ma, Ying Sun

**Affiliations:** Sun Yat-sen University Cancer Center; State Key Laboratory of Oncology in South China; Collaborative Innovation Center of Cancer Medicine, 651 Dongfeng Road East, Guangzhou, People’s Republic of China

**Keywords:** *ZNF671*, Hypermethylation, Proliferation, Tumorigenicity, Nasopharyngeal carcinoma

## Abstract

**Background:**

Epigenetic abnormalities play important roles in nasopharyngeal cancer (NPC), however, the epigenetic changes associated with abnormal cell proliferation remain unclear.

**Methods:**

We detected epigenetic change of *ZNF671* in NPC tissues and cell lines by bisulfite pyrosequencing. We evaluated zinc finger protein 671 (*ZNF671*) expression in NPC cell lines and clinical tissues using real-time PCR and western blotting. Then, we established NPC cell lines that stably overexpressed *ZNF671* and knocked down *ZNF671* expression to explore its function in NPC in vitro and in vivo. Additionally, we investigated the potential mechanism of *ZNF671* by identifying the mitotic spindle and G2/M checkpoint pathways pathway downstream genes using gene set enrichment analysis, flow cytometry and western blotting.

**Results:**

*ZNF671* was hypermethylated in NPC tissues and cell lines. The mRNA and protein expression of *ZNF671* was down-regulated in NPC tissues and cell lines and the mRNA expression could be upregulated after the demethylation agent 5-aza-2′-deoxycytidine treatment. Overexpression of *ZNF671* suppressed NPC cell proliferation and colony formation in vitro; silencing *ZNF671* using a siRNA had the opposite effects. Additionally, overexpression of *ZNF671* reduced the tumorigenicity of NPC cells in xenograft model in vivo. The mechanism study determined that overexpressing *ZNF671* induced S phase arrest in NPC cells by upregulating p21 and downregulating cyclin D1 and c-myc.

**Conclusions:**

Epigenetic mediated zinc finger protein 671 downregulation promotes cell proliferation and enhances tumorigenicity by inhibiting cell cycle arrest in NPC, which may represent a novel potential therapeutic target.

**Electronic supplementary material:**

The online version of this article (10.1186/s13046-017-0621-2) contains supplementary material, which is available to authorized users.

## Background

Nasopharyngeal carcinoma (NPC) is the most common head and neck cancer in Southern China and Southeast Asia [1]. Although local and regional control has improved since the introduction of intensity-modulated radiation therapy and chemoradiotherapy, approximately 30% of patients eventually develop recurrence and/or distant metastasis [2]. Therefore, improved understanding the molecular mechanisms that regulate NPC progression is essential to develop novel treatment strategies.

Uncontrolled proliferation is a pathological characteristic of cancer cells. Protein kinase complexes composed of cyclins and cyclin-dependent kinases (CDKs) determine the progression of cells through the cell cycle. Cyclins function as the regulatory subunit and CDKs function as the catalytic subunit of the activated heterodimer complexes, which orchestrate coordinated entry into the S phase of the cell cycle [3]. Dysregulation of cell cycle components can lead to uncontrolled tumor cell proliferation and cancer [4, 5]. Clinical trials targeting CDK inhibitors have shown promise for the treatment of cancer [6, 7]; therapeutic strategies targeting cell cycle-related proteins may be effective for the treatment of myeloma and breast cancer. However, the mechanisms leading to malignant proliferation in NPC remain poorly characterized.

DNA methylation is a critical epigenetic modification involved in regulation of gene expression [8]. Dysregulated methylation of specific genes has been shown to increase NPC cell growth, invasion and migration, and may contribute to the progression and recurrence of NPC [9–11]. In a previous study, we employed Illumina Human Methylation 450 K Beadchips to perform genome-wide DNA methylation analysis of 48 samples (between 24 nasopharyngeal carcinoma tissues and 24 normal nasopharyngeal epithelial tissues) to identify aberrantly methylated genes (GSE52068) [10]. One of the top-ranked hypermethylated genes, zinc finger protein 671 (*ZNF671*), which contains C2H2-type zinc fingers (ZFs) and a Krüppel associated box (KRAB) domain, is a member of the KRAB-ZFP family of mammalian transcriptional repressors [12, 13] that play important roles in regulation of cell differentiation, proliferation, apoptosis and tumor suppression [14, 15]. Recent studies have demonstrated that *ZNF671* is epigenetically silenced by DNA methylation and functions as a tumor suppressor in multiple carcinomas [16–18]. However, little is known about the function and mechanism of action of *ZNF671* in NPC.

Here, we report that *ZNF671* is downregulated and the *ZNF671* promoter is hypermethylated in NPC cell lines and tissues. Overexpression of *ZNF671* suppressed, while silencing *ZNF671* promoted, NPC cell proliferation and colony formation in vitro and tumorigenicity in vivo. Further studies demonstrated overexpression of *ZNF671* inhibited NPC cell proliferation and tumorigenicity by inducing S phase cell cycle arrest.

## Methods

### Cell culture and clinical specimens

Human NPC cell lines (CNE1, CNE2, HNE1, HONE1, SUNE1, 5-8F, 6-10B) were cultured in RPMI-1640 (Invitrogen, Life Technologies, Grand Island, NY) supplemented with 5% fetal bovine serum (FBS) (Gibco-BRL, Carlsbad, CA, USA). Human immortalized nasopharyngeal epithelial cell line (NP69, N2Tert) were cultured in keratinocyte serum-free medium (Invitrogen) supplemented with bovine pituitary extract (BD influx, Biosciences, USA). 293 T cells were obtained from the ATCC (Manassas, VA, USA) and maintained in DMEM (Invitrogen) supplemented with 10% FBS. Four freshly frozen NPC samples and four normal nasopharyngeal epithelium samples were collected from patients undergoing biopsy at Sun Yat-sen University Cancer Center.

### RNA isolation and reverse transcription-PCR (RT-PCR)

Total RNA was isolated from NPC cell lines using TRIzol Reagent (Invitrogen) following the manufacturer’s instructions, cDNA was synthesized using M-MLV reverse transcriptase (Promega, Madison, WI, USA) and amplified with Platinum SYBR Green qPCR SuperMix-UDG reagents (Invitrogen) using the CFX96 sequence detection system (Bio-Rad, Hercules, CA, USA) with the following primers: *ZNF671* forward, 5′- GACTTAGACCTGGTTGTTGG -3′ and reverse, 5′- GTATTTAGCCAGGTGTAAGGT-3′. *GAPDH* was used as control for normalization.

### Western blotting

RIPA lysis buffer (Beyotime, Shanghai, China) was used to isolate proteins and the Bradford method, to determine protein concentrations. Proteins (20 μg) were separated by SDS-polyacrylamide gel electrophoresis (SDS-PAGE, Beyotime), transferred onto PVDF membranes (Millipore, Billerica, MA, USA) and incubated with primary anti-*ZNF671* (1:500; Proteintech, Chicago, IL, USA), anti-cyclin D1 (1:1000; Cell Signaling Technology, Danvers, MA, USA), anti-c-myc (1:1000; Proteintech) or anti-p21 (1:1000; Proteintech) antibodies overnight at 4 °C, followed by species-matched secondary antibodies. Bands were detected using enhanced chemiluminescence.

### DNA isolation and bisulfite pyrosequencing analysis

NPC cell lines were treated with or without 10 μmol/L 5-aza-2′-deoxycytidine (DAC; Sigma-Aldrich, Munich, Germany) for 72 h, with the drug/media replaced every 24 h. DNA was isolated using the EZ1 DNA Tissue Kit (Qiagen, Hilden, Germany), then 1–2 μg DNA was treated with sodium bisulfite using the EpiTect Bisulfite kit (Qiagen) according to the manufacturer’s instructions. Bisulfite pyrosequencing primers were designed using PyroMark Assay Design Software 2.0 (Qiagen), and were: PCR forward primer: 5′-GAATTTAGGTTAGGGATAGTTTGAT-3′ (F); PCR reverse primer: 5′-CCAAAAAAAAAATATTTCAATACC-3′ (R); sequencing primer: 5′-GG ATAGTTTGA TAGAAATAAAATG-3′(S). The PyroMark Q96 System (Qiagen) was used for the sequencing reactions and to quantify methylation.

### Stable cell line establishment and ZNF671 small interfering RNAs (siRNAs)

The pSin-EF2-puro-*ZNF671*-HA or pSin-EF2-puro-vector plasmids were obtained from Land. Hua Gene Biosciences (Guangzhou, China); pSin-EF2-puro-Vector plasmid was used as a control. Stably transfected cells were selected using puromycin and confirmed using RT-PCR. SiRNAs targeting *ZNF671* were obtained from GenePharma Co., Ltd. (Shanghai, China); siRNA #1 targets *ZNF671*-Homo-626 cDNA (sense strand: CCUUACACCUGGCUAAAUATT; antisense strand: UAUUUAGCCAGGUGUAAGGTT) and siRNA #2 targets *ZNF671*-Homo-279 cDNA (sense strand: GGAAGAAUGGGAGCUUCUUTT; antisense strand: AAGAAGCUCCCAUUCUUCCTT).

### Cell proliferation and colony formation assays

For the CCK-8 assay, cells (1 × 10^3^) were seeded into 96-well plates, incubated for 0–4 days, stained with CCK-8 (Dojindo, Tokyo, Japan), and absorbance values were determined at 450 nm using a spectrophotometer. For the colony formation assay, cells (0.3 × 10^3^) were seeded into 6-well plates, cultured for 2 weeks and the colonies were fixed in methanol, stained with crystal violet and counted.

### Cell cycle analysis

Cells (2 × 10^5^) were seeded into 6-well plates, cultured for 24 h, serum-starved for 24 h to synchronize cells at the G1/S checkpoint, trypsinized, washed with ice-cold PBS, fixed in 70% ethanol, and stored at −20 °C until analysis. Before staining, cells were gently resuspended in cold PBS and RNase A was added into cell suspension tube incubated at 37 °C for 30 min, followed by incubation with propidium iodide (PI) (Beyotime) for 20 min at room temperature. The fluorescence intensity of the cells was analyzed by flow cytometry (Gallios; Beckman-Coulter, Germany).

### Animal experiments

BALB/c-nu mice (4–6 weeks old) were purchased from Charles River Laboratories (Beijing, China), and CNE2-vector or CNE2-*ZNF671* cells (1 × 10^6^) were subcutaneously inoculated into the dorsal flank. Tumor size was measured every 3 days and tumor volumes were calculated using the equation: volume = D × d^2^ × π/6, where D and d represent the longest and shortest diameters, respectively. All animal research was conducted in accordance with the detailed rules approved by the Animal Care and Use Ethnic Committee of Sun Yat-sen University Cancer Center and all efforts were made to minimize animal suffering.

### Gene set enrichment analysis (GSEA)

The GSEA software tool (version 2.0.13, www.broadinstitute.org/gsea/) was used to identify KEGG pathways (MSigDB, version 4.0) that show an overrepresentation of up- or downregulated genes between *ZNF671* high expression (*n* = 15) and *ZNF671* low expression (*n* = 16) in GSE12452. Briefly, an enrichment score was calculated for each gene set (i.e., KEGG pathway) by ranking each gene by their expression difference using Kolmogorov-Smirnov statistic, computing a cumulative sum of each ranked in each gene set, and recording the maximum deviation from zero as the enrichment score.

### Statistical analysis

Statistical analyses were performed using SPSS 17.0 (SPSS Inc., Chicago, IL, USA). All data shown are representative of at least three independent experiments, and values are expressed as the mean ± SD. Differences between two groups were analyzed using the two-tailed unpaired Student’s *t*-test; *p* < 0.05 was considered significant. All data from this study has been deposited at Sun Yat-sen University Cancer Center for future reference (number RDDB2017000075).

## Results

### The ZNF671 promoter is hypermethylated in NPC

**Fig. 1:**
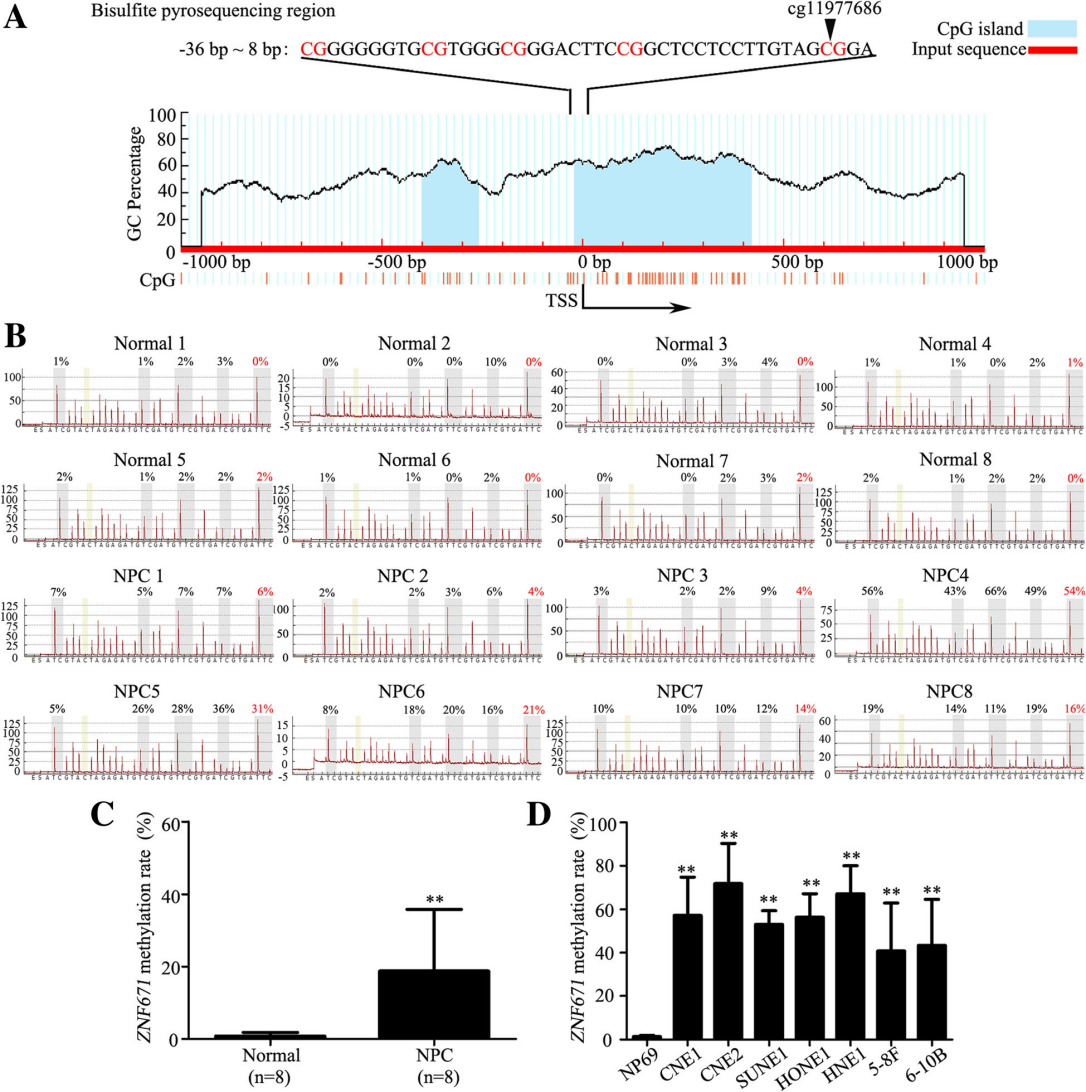
*ZNF671* is hypermethylated in NPC. **a** Schematic illustration of the *ZNF671* promoter CpG islands and bisulfite pyrosequencing region. Input sequence: red region; CpG islands: blue region; TSS: transcription start site; cg11977686: CG site identified in our previous genome-wide methylation analysis; red text: CG sites for bisulfite pyrosequencing; bold red text: most significantly altered CG site in *ZNF671*. **b** and **c** Bisulfite pyrosequencing analysis of the *ZNF671* promoter region (**b**) and the average methylation levels (**c**) in normal (*n* = 8) and NPC (n = 8) tissues. Red text: cg11977686 CG site. **d** Bisulfite pyrosequencing analysis of *ZNF671* promoter region, as determined by bisulfite pyrosequencing analysis, in NP69 and NPC (CNE1, CNE2, SUNE1, HONE1, HNE1, 5-8F and 6-10B) cell lines. **d** Quantitative RT-PCR analysis of *ZNF671* mRNA expression in NPC cell lines after DAC treatment. All experiments were performed at least three times; data are mean ± SD. **P < 0.01 vs. control, Student’s *t*-test

To confirm our previous methylation data (GSE52068) (Additional file 1: Figure S1A), the promoter methylation level of *ZNF671* was detected by bisulfite pyrosequencing analysis in other NPC (*n* = 8) and normal tissues (n = 8). The CpG islands and region selected for bisulfite pyrosequencing in the *ZNF671* promoter region are shown in Fig. 1a. The methylation of *ZNF671* (cg11977686) in NPC tissues were significantly increased compared with normal tissues (Fig. 1b and c). Similarly, *ZNF671* (cg11977686) methylation levels in the NPC cell lines (CNE1, CNE2, SUNE1, HONE1, HNE1, 5-8F and 6-10B) were also increased compared with human immortalized normal nasopharyngeal epithelial cell line (NP69) (Fig. 1d and Additional file 1: Figure S1B; *P* < 0.05). These results indicate that *ZNF671* promoter is hypermethylated in NPC.

### Promoter hypermethylation contributes to downregulation of ZNF671 in NPC

**Fig. 2:**
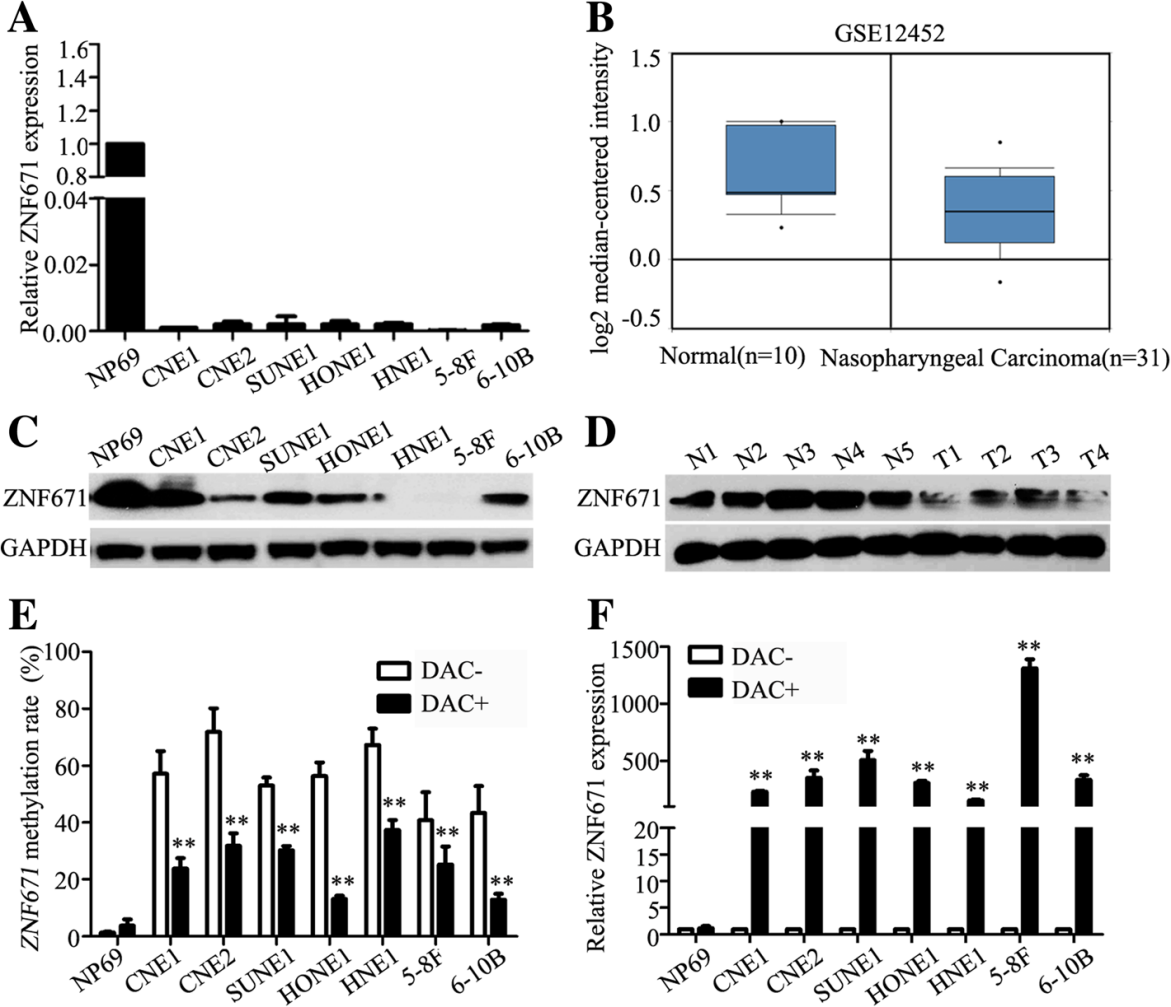
Promoter hypermethylation contributes to downregulation of ZNF671 in NPC. **a** Quantitative RT-PCR analysis of *ZNF671* mRNA expression in NP69 and NPC cell lines. **b** *ZNF671* mRNA is downregulated in the GSE12452 nasopharyngeal carcinoma dataset. **c-d** Western blotting analysis of *ZNF671* in NPC (CNE1, CNE2, SUNE1, HONE1, HNE1, 5-8F and 6-10B) cell lines and NPC (T, *n* = 4) and normal nasopharyngeal epithelial tissues (N, *n* = 4). **e** and **f** *ZNF671* methylation levels measured via bisulfite pyrosequencing analysis (**e**) and relative *ZNF671* mRNA levels measured via real-time RT–PCR analysis (**f**) with (DAC+) or without (DAC−) DAC treatment in NP69 and NPC cell lines. All experiments were repeated at least three times; data are mean ± SD; *P*-values were calculated using the Student’s *t*-test

To know the association between *ZNF671* expression and its promoter methylation status in NPC, quantitative RT-PCR revealed *ZNF671* mRNA was significantly downregulated in all seven NPC cell lines compared to normal nasopharyngeal epithelial NP69 cells (Fig. 2a). Analysis of microarray-based high-throughput NPC datasets (GSE12452) confirmed *ZNF671* was downregulated in NPC tissues compared to normal nasopharyngeal tissues (Fig. 2b; *P* < 0.05). Furthermore, western blotting showed *ZNF671* protein expression was downregulated in both the NPC cell lines and freshly frozen NPC tissues (*n* = 4) compared to normal samples (n = 4) (Fig. 2c and d; *P* < 0.05). To determine whether the downregulation of *ZNF671* results from its promoter hypermethylation, immortalized normal nasopharyngeal epithelial cell line and NPC cell lines were treated with or without the demethylation drug 5-aza-2′-deoxycytidine (Decitabine, DAC). The *ZNF671* methylation level were substantially decreased (Fig. 2e and Additional file 2: Figure S2; *P* < 0.05), while the *ZNF671* mRNA were significantly increased (Fig. 2f; *P* < 0.05) in NPC cell lines compared with immortalized normal nasopharyngeal epithelial cell. The findings suggest that *ZNF671* is downregulated in NPC and the downregulation of *ZNF671* is associated with the hypermethylation of *ZNF671* in NPC.

### ZNF671 suppresses NPC cell proliferation in vitro

**Fig. 3:**
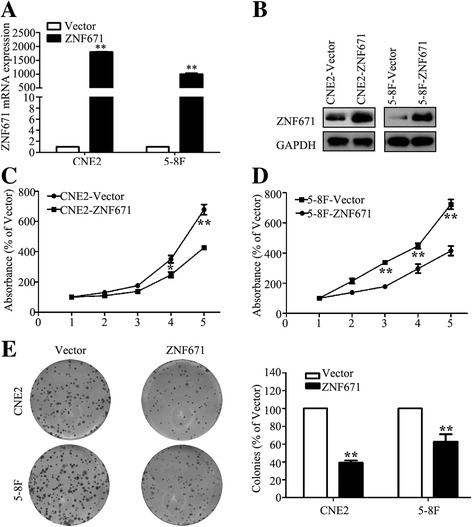
Effects of *ZNF671* overexpression on NPC cell viability and colony formation ability in vitro. **a** qPCR analysis of *ZNF671* mRNA expression in CNE-2 and 5-8F cells stably overexpression *ZNF671*. **b** Western blotting analysis of *ZNF671* expression in CNE-2 and 5-8F cells stably overexpression *ZNF671*. **c-d** The CCK-8 assay showed overexpression of *ZNF671* reduced the viability of CNE2 (**c**) and 5-8F (**d**) cells. **e** The colony formation assay showed overexpression of *ZNF671* suppressed colony-forming ability. All experiments were performed at least three times; data are mean ± SD. *P < 0.05, **P < 0.01 vs. control, Student’s *t-*test

To assess the effects of *ZNF671* on proliferation and metastasis in NPC, we subjected CNE2 and 5-8F cells stably overexpressing *ZNF671* or the control vector to the CCK8, colony formation and Transwell migration and invasion assays. As shown in Fig. 3a and b, qPCR and western blotting validated that *ZNF671* mRNA and protein level was obviously elevated after stably overexpressing *ZNF671* in NPC cells. The CCK8 assay demonstrated that the cell viability of CNE2 and 5-8F cells stably overexpressing *ZNF671* remarkably was much slower than that of cells expressing vector plasmid (Fig. 3c and d). Overexpressing *ZNF671* reduced the colony formation ability of CNE2 and 5-8F cells (Fig. 3e), but did not significantly affect cell migratory or invasive ability (Additional file 3: Figure S3). These findings indicate that *ZNF671* inhibits the proliferation abilities of NPC cells in vitro*.*

### Silencing ZNF671 promotes NPC cell proliferation in vitro

**Fig. 4:**
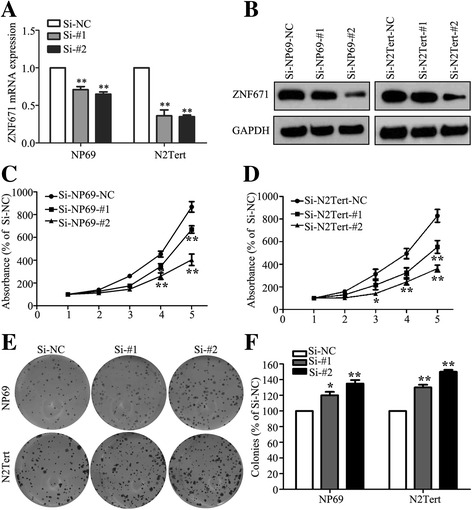
Effects of *ZNF671* silencing on NPC cell viability and colony formation in vitro. **a** qPCR analysis of *ZNF671* silence in NP69 and N2Tert cells. **b** Western blotting analysis of *ZNF671* silence in NP69 and N2Tert cells. **c-d** Silencing *ZNF671* increased the proliferation (**c**) and colony formation (**d**) ability of nasopharyngeal epithelial NP69 and N2Tert cells. All experiments were performed at least three times; data are mean ± SD. *P < 0.05, **P < 0.01 vs. control, Student’s *t-*test

To further investigate whether silencing of *ZNF671* affects the proliferation abilities of NPC cells, we transiently transfected NP69 and N2Tert cells with si*ZNF671* or control siRNA, and performed the CCK8 and colony formation assays. As shown in Fig. 4a and b, qPCR and western blotting confirmed that the *ZNF671* mRNA and protein level was remarkably decreased after silencing of *ZNF671* in NPC cells. The CCK8 assay that cells transfected with si*ZNF671* grew faster than cells transfected with control siRNA (Fig. 4c and d). Knocking down *ZNF671* promoted NPC cell colony formation capability as determined by the colony formation ability (Fig. 4e)*.* Collectively, these results indicate that silencing *ZNF671* promotes cell proliferation in NPC.

### ZNF671 inhibits tumorigenicity in an in vivo model of NPC

**Fig. 5:**
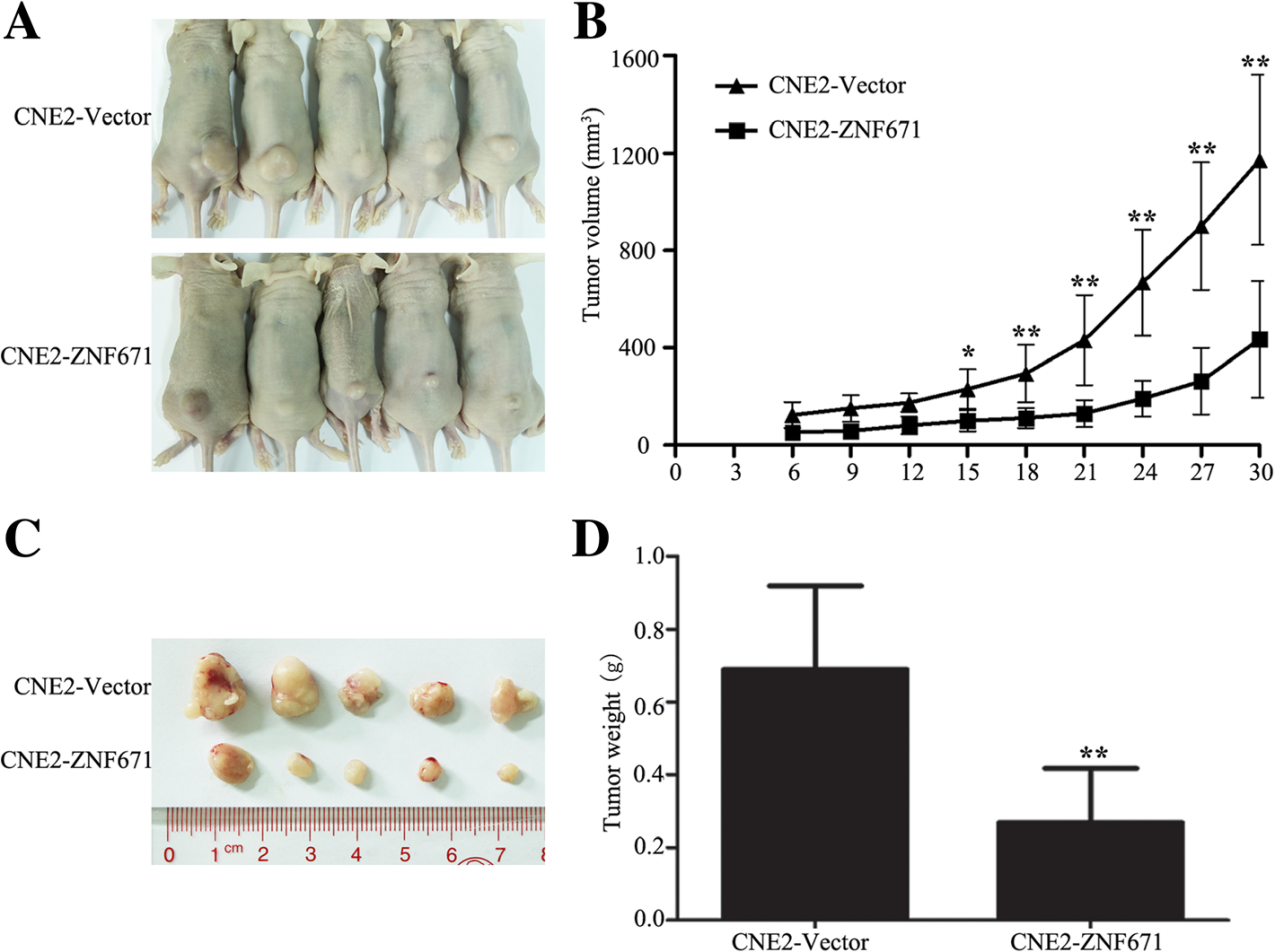
*ZNF671* reduces the tumorigenicity of NPC cells in vivo. **a** BALB/c mice were injected with the indicated cells. Images of the mice and tumors formed at 30 days after injection. **b** Growth curves of tumor volume. **c** Images of the excised tumors. **d** Excised tumor weight. Data are mean ± SD. *P < 0.05, **P < 0.01 vs. control, Student’s *t*-test

Next, the effect of *ZNF671* on the tumorigenicity of human NPC cells was examined in vivo. As shown in Fig. 5a and b, the tumors in the group injected with cells stably overexpressing *ZNF671* grew at a slower rate and had smaller volumes than the vector control tumors. When the mice were sacrificed on day 30, the tumors formed by *ZNF671*-overexpressing cells were significantly lighter than vector control tumors (0.69 ± 0.18 g vs. 0.27 ± 0.14 g; *P < 0.05, **P < 0.01; Fig. 5c and d). Taken together, these results indicate that downregulation of *ZNF671* enhances the tumorigenicity of NPC cells in vivo.

### ZNF671 inhibits NPC cell proliferation by inducing S phase cell cycle arrest

**Fig. 6:**
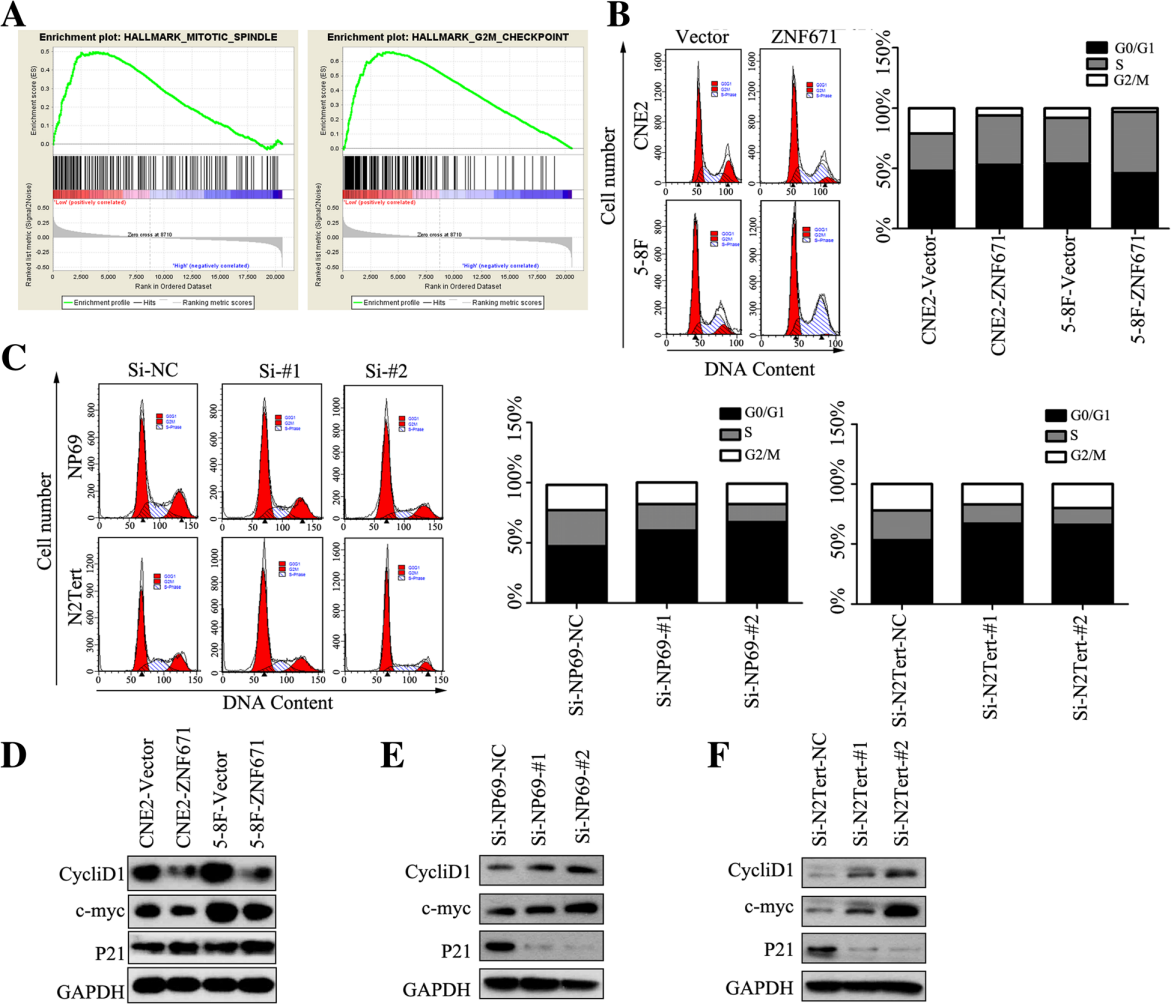
*ZNF671* induces cell cycle arrest at the S phase. **a** GSEA enrichment plots revealed that enrichment of mitotic spindle and G2/M checkpoint pathways was associated with downregulation of *ZNF671*. **b-c** Flow cytometry analysis of cell cycle distribution in cells overexpressing *ZNF671* and (**c**) after silencing *ZNF671*. **d**-**f** Western blot analysis of proteins related to the S phase in cells overexpressing *ZNF671* and (**e**-**f**) after silencing *ZNF671*

To further investigate the mechanism by which *ZNF671* inhibits cell proliferation in NPC, we performed gene set enrichment analysis (GSEA) in the GEO database to identify pathways potentially linked to *ZNF671*. As shown in Fig. 6a, pathways related to hallmarks of the mitotic spindle and G2/M checkpoint genes were enriched in GSE12452 database. Indeed, we observed enrichment of gene sets associated with cancer, including the mitotic spindle and G2/M checkpoint pathways, in *ZNF671*-high expressing tumors; conversely, these pathways were not enriched in *ZNF671*-low expressing tumors.

Flow cytometry confirmed that overexpression of *ZNF671* significantly decreased the percentage of cells in the G2/M phase (20.67% ± 0.34% vs. 6.40% ± 0.22% in CNE2 cells, 8.47% ± 1.20% vs. 4.04% ± 0.56% in 5-8F cells; *P* < 0.05) and increased the proportion of cells in the S phase (30.88% ± 0.12% vs. 41.12% ± 0.28% in CNE2 cells, 36.64% ± 0.92% vs. 51.94% ± 0.23% in 5-8F cells; *P* < 0.05; Fig. 6b). Conversely, silencing *ZNF671* decreased the percentage of cells in the S phase (from 27.92% ± 4.3% to 14.05% ± 0.73% and 20.29% ± 1.6% in NP69-Si#1 and NP69-Si#2 cells, and from 25.21% ± 0.10% to 17.69% ± 4.32% and 16.18% ± 1.38% in N2Tert-Si#1 and N2tert-Si#2 cells; Fig. 6c; *P* < 0.05). Furthermore, bioinformatics analysis indicated that *ZNF671* is involved in regulation of the cycle-related proteins cyclin D1 and p21. Overexpression of *ZNF671* decreased the protein levels of cyclin D1 and c-myc, and increased p21 (Fig. 6d). Additionally, silencing *ZNF671* increased the expression of cyclin D1 and c-myc and decreased the expression of p21 (Fig. 6e and f). Taken together, these data indicate that downregulation of *ZNF671* in NPC promotes cell proliferation by preventing S phase cell cycle arrest.

## Discussion

This study demonstrates *ZNF671* is downregulated in NPC, consistent with our previous analysis of publicly available NPC datasets [13], due to promoter hypermethylation. Moreover, overexpressing *ZNF671* reduced NPC cell viability and colony formation in vitro, enhanced tumorigenicity in vivo*,* and induced cell cycle arrest. These results provide new mechanistic understanding of the ability of *ZNF671* to regulate cell proliferation in NPC.

Local recurrence and distant metastasis are the major patterns of treatment failure in NPC. Most cancers are caused by accumulation of genomic or epigenetic alterations [19–24], and epigenetic alterations play an important role in the development of NPC [9, 25]. Several studies have indicated that aberrantly methylated genes could serve as prognostic biomarkers for NPC [26–28]. Thus, exploration of the mechanisms by which gene methylation contributes to progression and recurrence are important strategies for improving prognosis and designing targeted therapies for NPC.

*ZNF671*, a member of the KRAB-ZF family, is silenced by promoter methylation in renal cell, cervical carcinoma and urothelial carcinoma [16, 18, 29]. A number of ZNF proteins function as tumor suppressors, and are epigenetically silenced by DNA methylation in multiple human cancers [14, 30]. We demonstrated *ZNF671* mRNA and protein expression are downregulated in NPC cell lines and tissues. Furthermore, overexpression of *ZNF671* suppressed NPC cell viability and colony formation in vitro and reduced tumorigenicity in vivo. These findings indicate that *ZNF671* functions as a tumor suppressor in NPC, consistent with its role in urothelial carcinoma [16].

Several individual components of the cell cycle machinery are subject to aberrant methylation, which contributes to malignancy in several cancers [31–33]. The S phase cell cycle check point ensures synthesis of DNA and proteins and is a crucial regulator of cell cycle progression, while the G2/M checkpoint allows cells to enter mitosis [34, 35]. Cyclin D1 and p21 cyclin-dependent kinases are major regulators of S phase progression [36–38]. Recent studies showed the transcriptionally repressive ZNF-KRAB domain can recruit KRAB-associated protein-1 (KAP1) [12, 13, 39] and other co-repressors, and KRAB-ZFP forms heterochromatin with chromobox 5 (CBX5), SET domain bifurcated 1 (SETDB1) and various histone deacetylases (HDACs) to epigenetically silence KRAB-ZNF target genes [40–42]. Our bioinformatic analysis showed *ZNF671* affects NPC cell proliferation by regulating the mitotic spindle and G2/M checkpoint pathways. Flow cytometry and western blotting confirmed overexpression of *ZNF671* induced S phase cell cycle arrest and blocked G2/M phase progression by downregulating cyclin D1 and c-myc and upregulating p21.

## Conclusions

The potential tumor suppressor *ZNF671* is epigenetically silenced by promoter methylation in NPC. Downregulation of *ZNF671* promotes NPC cell proliferation and tumorigenicity by facilitating cell cycle progression. These findings provide new insight into the molecular mechanisms that regulate NPC progression and may help to identify novel therapeutic targets and strategies.

## Additional files

Additional file 1: Figure S1. *ZNF671* is hypermethylated in NPC. (A) Methylation levels of *ZNF671* in Normal (*n* = 24) and NPC (n = 24) tissues from the genome-wide methylation microarray data. Mean ± ± SD; Student’s t-tests. (B) Bisulfite pyrosequencing analysis of the *ZNF671* promoter region in NP69 and NPC (CNE1, CNE2, SUNE1, HONE1, HNE1, 5-8F and 6-10B) cell lines. Red words: CG site of cg11977686. *P < 0.05, **P < 0.01 vs. control, Student’s *t*-test. (TIFF 831 kb)

Additional file 2: Figure S2. *ZNF671* is hypermethylated in NPC cells. Bisulfite pyrosequencing analysis of the *ZNF671* promoter region in NP69 and NPC (CNE1, CNE2, SUNE1, HONE1, HNE1, 5-8F and 6-10B) cell lines following treatment with DAC. Red words: CG site of cg11977686. *P < 0.05, **P < 0.01 vs. control, Student’s *t*-test. (TIFF 861 kb)

Additional file 3: Figure S3. *ZNF671* has no effect on affect NPC migratory and invasive ability. (A) Migration ability was measured using a wound healing assay (200 ×) and (B) Transwell assay with Matrigel (200 ×) in CNE2 and SUNE1 cells with the vector or *ZNF671* overexpression. Scale bar: 100 μm; data are mean ± SD. *P < 0.05, **P < 0.01 vs. control, Student’s *t-*test. (TIFF 1066 kb)

## Abbreviations

CCK-8: Cell counting kit-8
DAC: 5-aza-2′-deoxycytidine
FBS: Fetal bovine serum
GSEA: Gene set enrichment analysis
HDAC: Histone deacetylase
NPC: Nasopharyngeal carcinoma
PAGE: Polyacrylamide gel electrophoresis
PBS: Phosphate-buffered saline
PI: Propidium iodide
RT-PCR: Real-time polymerase chain reaction
siRNA: Small interfering RNA
