# Correction to: Epigenetic mediated zinc finger protein 671 downregulation promotes cell proliferation and tumorigenicity in nasopharyngeal carcinoma by inhibiting cell cycle arrest

**DOI:** 10.1186/s13046-021-02205-0

**Published:** 2021-12-15

**Authors:** Jian Zhang, Xin Wen, Na Liu, Ying-Qin Li, Xin-Ran Tang, Ya-Qin Wang, Qing-Mei He, Xiao-Jing Yang, Pan-Pan Zhang, Jun Ma, Ying Sun

**Affiliations:** Sun Yat-sen University Cancer Center; State Key Laboratory of Oncology in South China; Collaborative Innovation Center of Cancer Medicine, 651 Dongfeng Road East, Guangzhou, People’s Republic of China

**Correction to: J Exp Clin Cancer Res 36, 147 (2017)**

**https://doi.org/10.1186/s13046-017-0621-2**

Following publication of the original article [1], the authors identified some minor errors in Fig. 3 and Fig. S3, specifically:

- Figure 3e: incorrect images were used for colony formation assay images for CNE2 Vector, CNE2 ZNF671 and 5-8F ZNF671 (top left, top right, and bottom right)
- Fig. S3a: incorrect images were used for migration assay images for CNE2 ZNF671 at both 0 h and 48 h (left panel, bottom row)

**Fig. 3:**
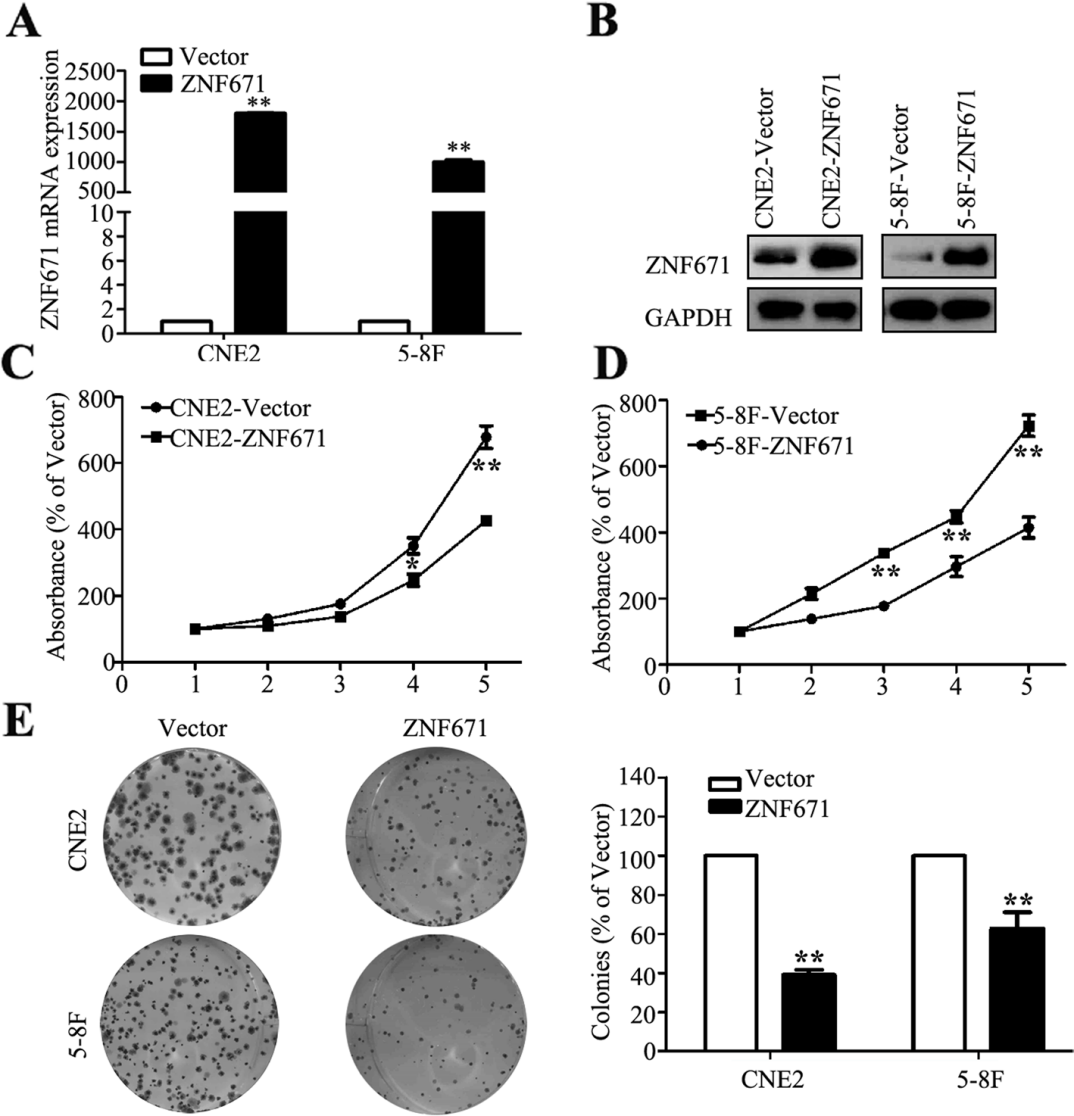
Effects of *ZNF671* overexpression on NPC cell viability and colony formation ability in vitro. **a** qPCR analysis of *ZNF671* mRNA expression in CNE-2 and 5-8F cells stably overexpression *ZNF671*. **b** Western blotting analysis of *ZNF671* expression in CNE-2 and 5-8F cells stably overexpression ZNF671. **c-d** The CCK-8 assay showed overexpression of *ZNF671* reduced the viability of CNE2 (**c**) and 5-8F (**d**) cells. **e** The colony formation assay showed overexpression of *ZNF671* suppressed colony-forming ability. All experiments were performed at least three times; data are mean ± SD. *P < 0.05, **P < 0.01 vs. control, Student’s t-test

The corrected figures are given here. The corrections do not have any effect on the final conclusions of the paper. The original article has been corrected.

## Supplementary Information

**Additional file 3 Fig. S3.** *ZNF671* has no effect on affect NPC migratory and invasive ability. (A) Migration ability was measured using a wound healing assay (200 ×) and (B) Transwell assay with Matrigel (200 ×) in CNE2 and SUNE1 cells with the vector or *ZNF671* overexpression. Scale bar: 100 μm; data are mean ± SD. *P < 0.05, **P < 0.01 vs. control, Student’s t-test.
