# Supplementary figures and images for: Epigenetic mediated zinc finger protein 671 downregulation promotes cell proliferation and tumorigenicity in nasopharyngeal carcinoma by inhibiting cell cycle arrest
Source: J Exp Clin Cancer Res. 2017 Oct 19;36:147. doi: 10.1186/s13046-017-0621-2 (PMC5649082; doi:10.1186/s13046-017-0621-2)

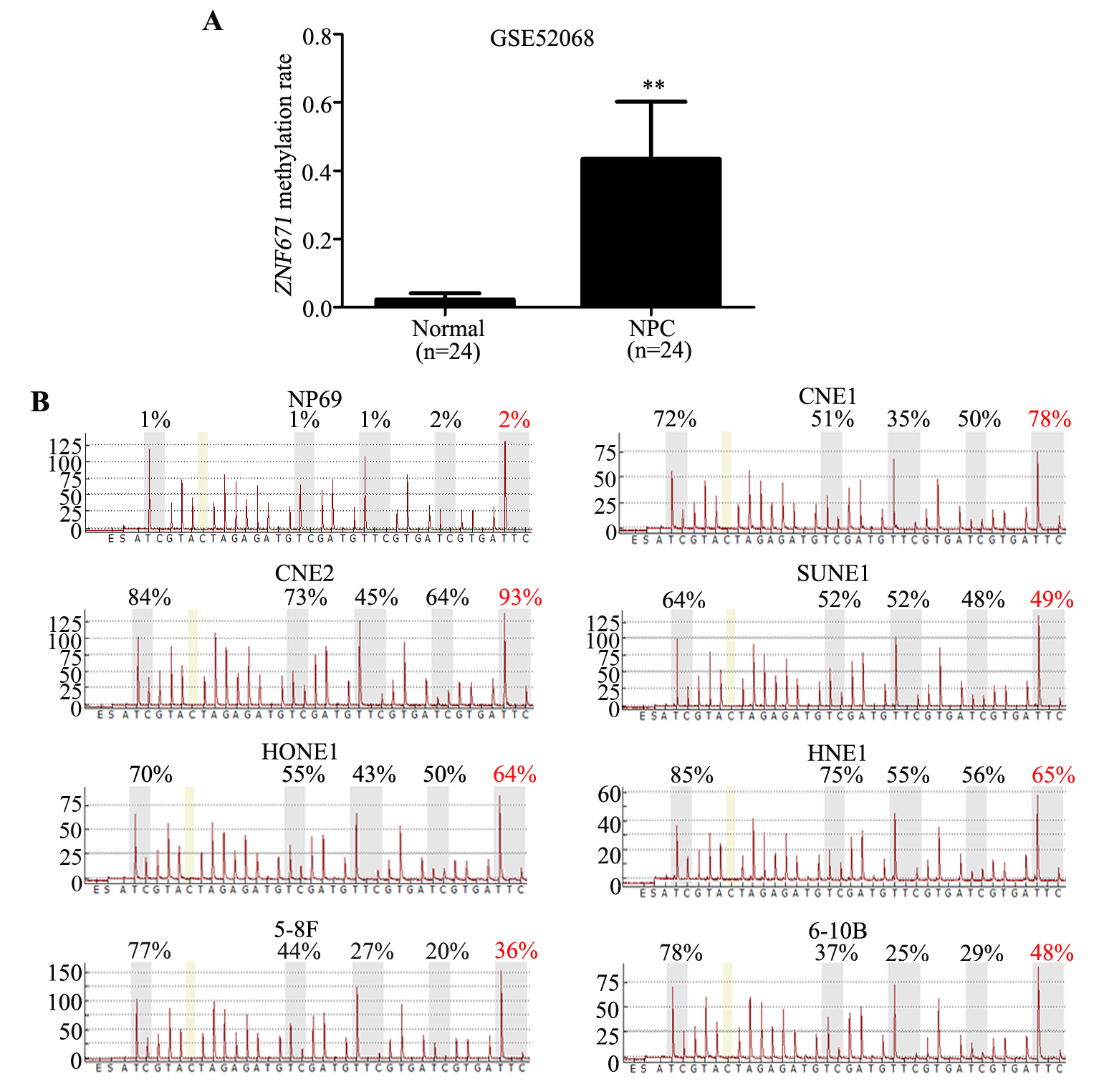

Supplement: Supplementary file 1 — ZNF671 is hypermethylated in NPC. (A) Methylation levels of ZNF671 in Normal (n = 24) and NPC (n = 24) tissues from the genome-wide methylation microarray data. Mean ± ± SD; Student’s t-tests. (B) Bisulfite pyrosequencing analysis of the ZNF671 promoter region in NP69 and NPC (CNE1, CNE2, SUNE1, HONE1, HNE1, 5-8F and 6-10B) cell lines. Red words: CG site of cg11977686. *P < 0.05, **P < 0.01 vs. control, Student’s t-test. (TIFF 831 kb) [file 13046_2017_621_MOESM1_ESM.tif]

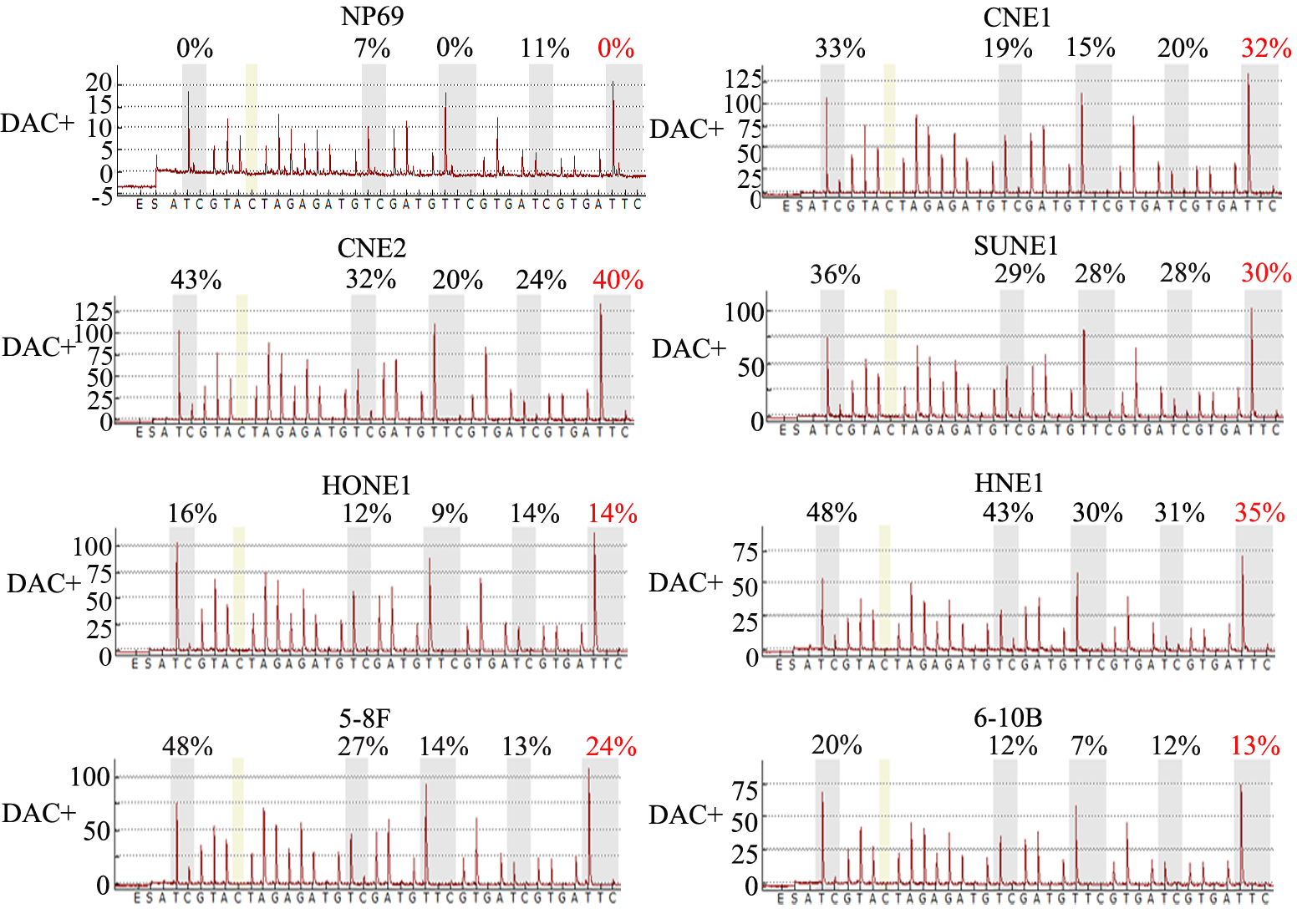

Supplement: Supplementary file 2 — ZNF671 is hypermethylated in NPC cells. Bisulfite pyrosequencing analysis of the ZNF671 promoter region in NP69 and NPC (CNE1, CNE2, SUNE1, HONE1, HNE1, 5-8F and 6-10B) cell lines following treatment with DAC. Red words: CG site of cg11977686. *P < 0.05, **P < 0.01 vs. control, Student’s t-test. (TIFF 861 kb) [file 13046_2017_621_MOESM2_ESM.tif]

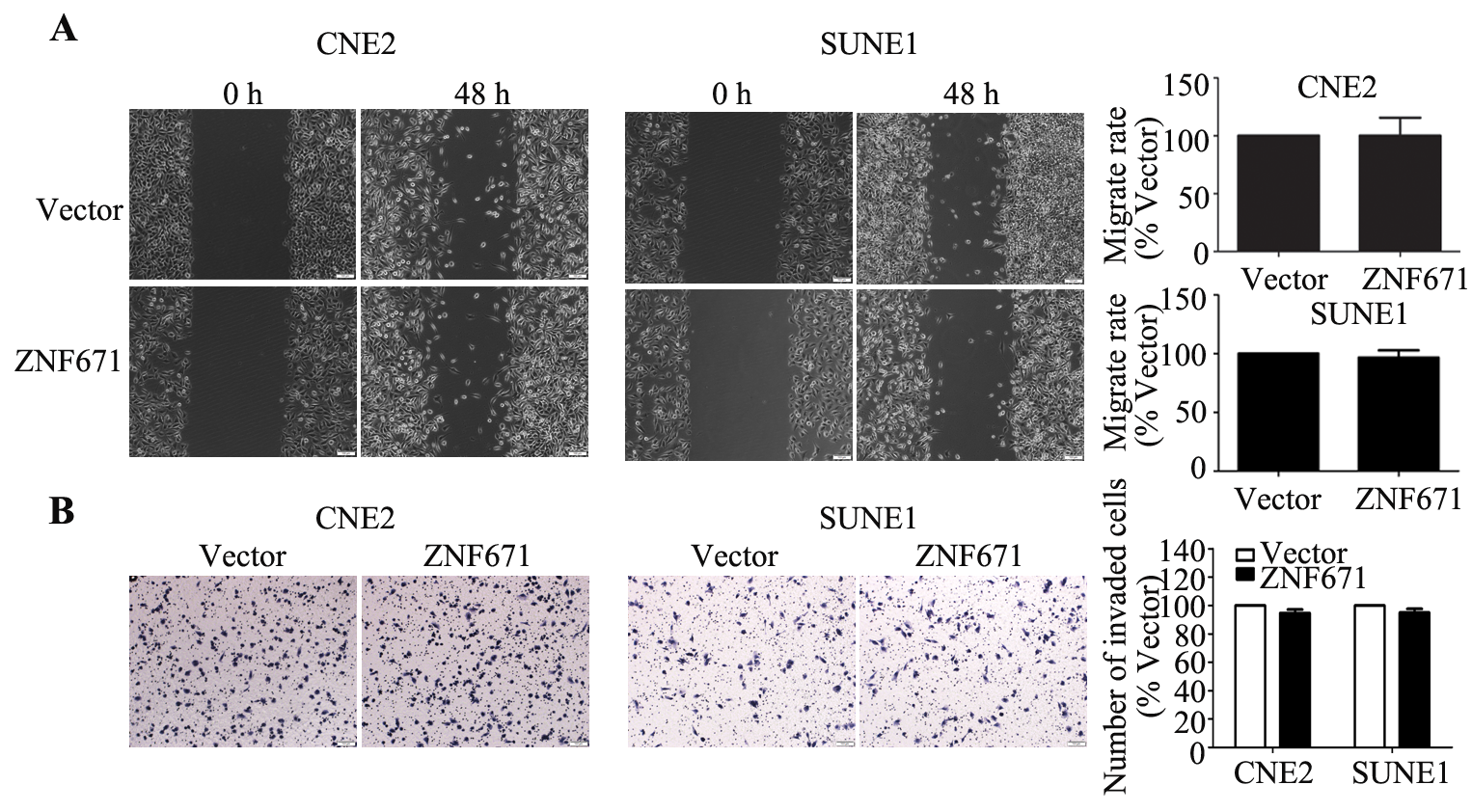

Supplement: Supplementary file 3 — ZNF671 has no effect on affect NPC migratory and invasive ability. (A) Migration ability was measured using a wound healing assay (200 ×) and (B) Transwell assay with Matrigel (200 ×) in CNE2 and SUNE1 cells with the vector or ZNF671 overexpression. Scale bar: 100 μm; data are mean ± SD. *P < 0.05, **P < 0.01 vs. control, Student’s t-test. (TIFF 1066 kb) [file 13046_2017_621_MOESM3_ESM.tif]
