# Supplementary figures and images for: Correction to: Epigenetic mediated zinc finger protein 671 downregulation promotes cell proliferation and tumorigenicity in nasopharyngeal carcinoma by inhibiting cell cycle arrest
Source: J Exp Clin Cancer Res. 2021 Dec 15;40:394. doi: 10.1186/s13046-021-02205-0 (PMC8672624; doi:10.1186/s13046-021-02205-0)

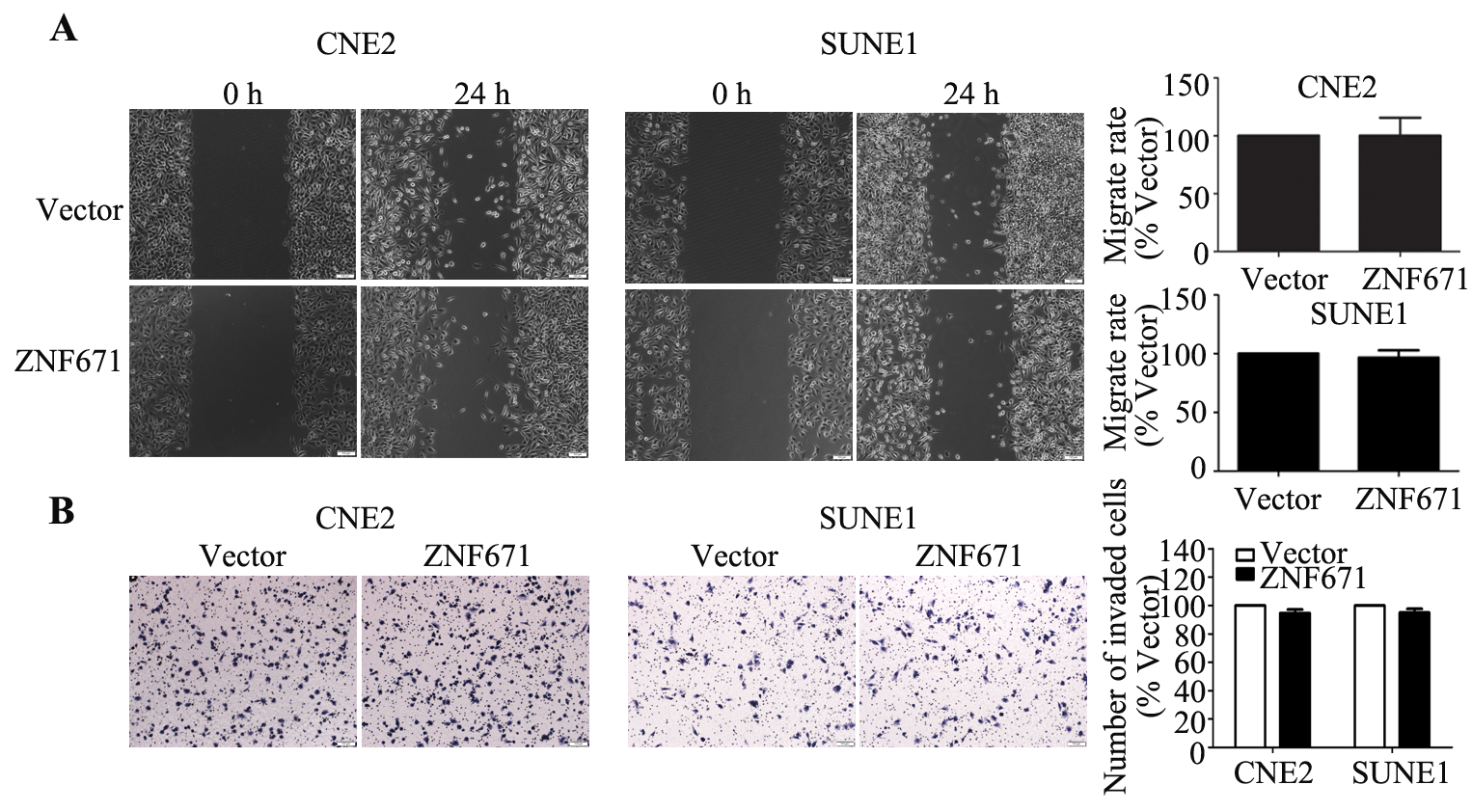

Supplement: Supplementary file 1 — Additional file 3 Fig. S3. ZNF671 has no effect on affect NPC migratory and invasive ability. (A) Migration ability was measured using a wound healing assay (200 ×) and (B) Transwell assay with Matrigel (200 ×) in CNE2 and SUNE1 cells with the vector or ZNF671 overexpression. Scale bar: 100 μm; data are mean ± SD. *P < 0.05, **P < 0.01 vs. control, Student’s t-test. [file 13046_2021_2205_MOESM1_ESM.tif]
